# Alcohol-Associated Pancreatitis and Liver Disease Among Adolescents and Young Adults

**DOI:** 10.1001/jamanetworkopen.2024.61990

**Published:** 2025-02-27

**Authors:** Oril Chapman, Maya Djerboua, Mandip Rai, Robert Bechara, Jennifer A. Flemming

**Affiliations:** 1 Department of Medicine, Queen’s University, Kingston, Ontario, Canada; 2 ICES Queen’s, Queen’s University, Kingston, Ontario, Canada; 3 Department of Public Health Sciences, Queen’s University, Kingston, Ontario, Canada

## Abstract

This cohort study examined the annual incidence rates of alcohol-associated end-organ disease among adolescents and young adults in Ontario, Canada, from 2003 to 2021.

## Introduction

Alcohol is the cause of deaths among 13.5% of individuals aged 20 to 39 years worldwide.^1^ Studies suggest the incidence of alcohol-associated liver disease (ALD) is increasing disproportionally among adolescents and young adults (AYAs), especially young females.^2,3,4^ Alcohol is also a main cause of acute and chronic pancreatitis and can affect other organs, such as the heart and stomach.^5^ However, the epidemiology of alcohol-associated end-organ complications other than ALD among AYAs has not been described. This study aimed to describe the epidemiology of end-organ complications from alcohol among AYAs over the past 2 decades in the general population.

## Methods

This retrospective, population-based cohort study from Ontario, Canada used routinely collected administrative health care data from ICES between 2003 and 2021. Incident emergency department or inpatient encounters for end-organ alcohol-related harm in AYAs aged 13 to 39 years were identified. The encounters were categorized by organ: (1) pancreas (alcohol-associated acute and chronic pancreatitis); (2) liver (ALD, alcohol-associated hepatitis, alcohol-associated cirrhosis); and (3) other organs (stomach, adrenal glands, nervous system, muscles, heart, and fetus). Age-adjusted annual incidence rates (IRs) of alcohol-related encounters were calculated per 100 000 person-years (PYs) and stratified by organ type and sex. Changes in annual rates by sex and organ were evaluated using Poisson regression and rate ratios (RRs) (Supplement 1). The Queen’s University Health Sciences Research Ethics Board approved this study and waived informed consent because deidentified data were used. We followed the STROBE reporting guideline.

Data were analyzed from month year to month year using SAS Enterprise Guide 7.1 (SAS Institute Inc). Two-sided *P* < .05 was considered significant.

## Results

In total, 11 508 AYAs with an incident end-organ complication from alcohol were identified (Table). Most were male AYAs (7366 [64%]), with a median IQR age of 28 (22-34) years. Pancreas-related complications were more frequent than liver-related complications (29% vs 19% respectively). Most pancreatic complications were acute pancreatitis (92%). Compared with AYAs with other end-organ complications, those with pancreas-related complications were more likely to be male (71%), reside in urban locations (88%), and require hospitalization (77%).

**Table.** Baseline Demographics of Adolescents and Young Adults (AYAs) With End Organ Complications Related to Alcohol in Ontario From 2003 to 2021

Demographic	AYAs, No. (%)			
	Overall (N = 11 508)	With pancreatic complications (n = 3310)	With liver complications (n = 2217)	With other organ complications (n = 5981)
Age, median (IQR)	28 (22-34)	31 (26-35)	33 (29-37)	24 (20-30)
Sex				
Female	4142 (36)	969 (29)	822 (37)	2351 (39)
Male	7366 (64)	2341 (71)	1395 (63)	3630 (61)
Residence				
Urban	9533 (83)	2910 (88)	1865 (84)	4758 (80)
Rural	1938 (17)	391 (12)	344 (16)	1203 (20)
Missing data	37 (<1)	9 (<1)	8 (<1)	20 (<1)
Income quintile				
1 (lowest)	3368 (29)	910 (28)	684 (31)	1774 (30)
2	2415 (21)	714 (22)	445 (20)	1256 (21)
3	2101 (18)	642 (19)	391 (18)	1068 (18)
4	1853 (16)	540 (16)	373 (17)	940 (16)
5 (highest)	1629 (14)	481 (15)	299 (13)	849 (14)
Missing data	142 (1)	23 (<1)	23 (<1)	94 (2)
Type of encounter				
ED only	7303 (63)	747 (23)	1010 (46)	5546 (93)
Inpatient	4205 (37)	2563 (77)	1207 (54)	435 (7)

Abbreviation: ED, emergency department.

The Figure shows annual age-adjusted IRs of end-organ alcohol harm per 100 000 PYs stratified by organ and sex. Overall, the incidence of pancreatitis increased by 7% per year in males (RR, 1.07; 95% CI, 1.06-1.08) and 12% per year in females (RR, 1.12; 95% CI, 1.10-1.13), with liver-related complications increasing by 6% per year in males (RR, 1.06; 95% CI, 1.05-1.07) and 9% per year in females (RR, 1.09; 95% CI, 1.08-1.11). Rates of end-organ alcohol harm in other organs decreased by 1% per year in males (RR, 0.99; 95% CI, 0.98-0.99) and increased by 2% per year in females (RR, 1.02; 95% CI, 1.01-1.02).

**Figure.**
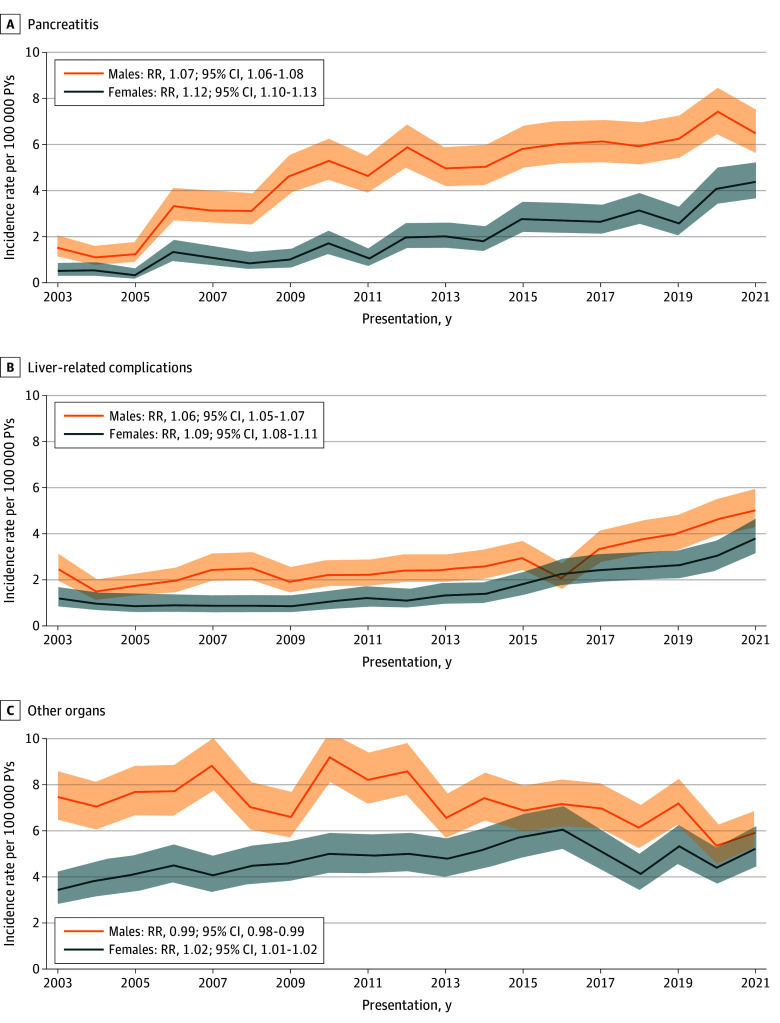
Incidence Rates per Person-Years (PYs) of Alcohol-Related End Organ Harms in Adolescents and Young Adults in Ontario From 2003-2021 Stratified by Organ and Sex RR indicates rate ratio.

## Discussion

Our findings suggest that gastrointestinal complications from alcohol are increasing in AYAs at rates much higher than in other organ systems. Males were most affected by both pancreatitis and ALD, yet young females had changes in the rates of alcohol-associated pancreatitis and ALD that were higher than males. To our knowledge, this is the first study to describe the epidemiology of alcohol-associated pancreatitis among AYAs. Similar to ALD, males and females may have a different risk of acute and chronic pancreatitis for the same level of alcohol exposure.^6^ It is unclear what factors are associated with this rise in disease burden. Although changes to administrative coding or better case definition could be contributing factors, we did not observe these changes in other end organs, such as the stomach and heart.

Study limitations include a lack of data on the quantity of alcohol consumption and the severity of disease at presentation. These findings underscore the importance of gastroenterologists in caring for individuals with harmful alcohol use and support the integration of addiction medicine into gastroenterology training along with consideration for multidisciplinary gastroenterology-addiction medicine clinics. Future research to understand these observations and evaluations in other age groups is urgently needed.

## Notes

Supplement 1.
eMethods

Supplement 2.
Data Sharing Statement
