# Supplementary material for: Alcohol-Associated Pancreatitis and Liver Disease Among Adolescents and Young Adults
Source: JAMA Netw Open. 2025 Feb 27;8(2):e2461990. doi: 10.1001/jamanetworkopen.2024.61990 (PMC11868968; doi:10.1001/jamanetworkopen.2024.61990)
Supplement: Supplement 1. — eMethods [file jamanetwopen-e2461990-s001.pdf]

Supplemental Online Content

Chapman O, Djerboua M, Rai M, Bechara R, Flemming JA. Incidence of Alcohol-Associated Pancreatitis and Liver Disease Among Adolescents and Young Adults. *JAMA Netw Open*. 2025;8(2):e2461990. doi: 10.1001/jamanetworkopen.2024.61990

eMethods

This supplemental material has been provided by the authors to give readers additional information about their work.

© 2025 Chapman O et al. *JAMA Network Open*.

eMethods

Databases

We conducted a population-based retrospective cohort study in Ontario, Canada from January 1, 2003 to December 31, 2021. This study utilized data from ICES (www.ices.on.ca), an independent, non-profit research institute that houses a large electronic repository of datasets, including over 20 years of routinely collected administrative health data at the individual level for all Ontario residents who are eligible for the province's single payer healthcare system, the Ontario Health Insurance Plan (OHIP). ICES receives funding and support by the Ministry of Health and Long-term Care and operates as a prescribed entity under Section 45 of Ontario's Personal Health Information Privacy Act (PHIPA), which enables ICES to use and analyze health data for the purposes of healthcare system evaluation and planning. Personal health records are stripped of identifiable or personal information and individuals are assigned a unique identifier to ensure anonymity and privacy protection prior to data analysis.

This study utilized the following administrative datasets: a) data for hospital admissions (the Canadian Institute for Health Information [CIHI] Discharge Abstract Database [DAD]); b) emergency department visits (the National Ambulatory Care Reporting System [NACRS]); c) demographics (the Registered Persons Database [RPDB]); and e) income quintile (Postal Code Conversion Files [PCCF]). These datasets were linked using unique encoded identifiers and analyzed at the ICES-Queen's Site.

Ethics

© 2025 Chapman O et al. *JAMA Network Open*.

The study protocol received ethics approval from the Queen's University Health Sciences Research Ethics Board (DMED 2624-21). The study was conducted in accordance with the Strengthening the reporting of observation studies in epidemiology (STROBE) guidelines for observational studies.

Study Cohort and Demographics

To calculate annual incidence rates of end-organ damage from alcohol, a cohort of AYAs 13-39 years of age residing in Ontario from 2003-2021 considered at risk for alcohol-related end organ complications were identified from the Registered Persons Database (RPDB). We considered AYAs to be at risk if they had no previous healthcare encounter for our outcomes of interest any time prior to cohort entry which occurred from the time that they turned 13 years old, or study start date January 1, 2003 if they were already between 13-39 years of age at that time. We defined the outcomes of end-organ complications secondary to alcohol using International Classification of Diseases 10th (ICD-10) codes if found as the main/primary diagnosis from emergency department (ED) or hospitalizations identified through inpatient hospital admissions (DAD) and ED records (NACRS). We used the following ICD-10 codes to identify the organ system affected:

	**ICD-10**
**Pancreas**	K852 (alcohol-associated acute pancreatitis)<br>K860 (alcohol-associated chronic pancreatitis)
**Liver**	K700 (alcoholic fatty liver)<br>K701 (alcoholic hepatitis)<br>K702 (alcoholic fibrosis and sclerosis of the liver)<br>K703 (alcoholic cirrhosis)<br>K709 (alcoholic liver disease unspecified)

© 2025 Chapman O et al. *JAMA Network Open*.

**Other end organs**	E244 (Adrenal gland)<br>G312, G621 (Neurologic system)<br>G721 (Muscles)<br>I426 (Heart)<br>K292 (Stomach)<br>P043, Q860, Q99304, Q99305 (Fetus)

The lookback window to exclude prevalent disease was for the entirety of the database holdings (1988 for DAD and 2000 for NACRS) which provided at least 3 years of lookback for all individuals for both inpatient and ED encounters. AYAs were considered no longer "at-risk" and were censored at: 1) the time they have the outcome of interest (alcohol-related end-organ complication); 2) turned 40 years of age; 3) died; 4) lost OHIP coverage or; 5) the end of the study period (December 31, 2021).

Among those AYAs who developed an outcome during the study period, they were further described stratified by type of end-organ by age and sex (RPDB), urban vs. rural residence based on communities +/- 10,000 inhabitants and income quintile based on linkage of the individual's postal code in RPDB to the PCCF for the Census-based relative household income at the dissemination area-level. Those with missing values for the above were also described.

<u>Statistical Analysis</u>

Age-adjusted annual incidence rates of end-organ damage from alcohol among AYAs stratified by organ type and sex were calculated per 100,000 person years (PY) with 95% confidence intervals. Next, Poisson regression was used to evaluate differences in annual IR stratified by sex and organ type to generate annual rate ratios and 95% confidence intervals. All statistical tests

© 2025 Chapman O et al. *JAMA Network Open*.

were evaluated at a significance level of $P < 0.05$. All data were analyzed using SAS Enterprise Guide Version 7.1 (SAS Institute Inc., Cary, North Carolina, U.S.A.).

© 2025 Chapman O et al. *JAMA Network Open*.
